# Efficacy and safety of a navigation-assisted, multi-approach endoscopic surgical strategy for orbital apex cavernous hemangiomas

**DOI:** 10.3389/fonc.2026.1761234

**Published:** 2026-07-22

**Authors:** Pei Wang, Xiuhong Li, Zexi Sang, Ru Lin, Yan Xun, Jiantao Xu, Hongfeng Yuan

**Affiliations:** 1 Department of Orbital Surgery, Chongqing Aier Eye Hospital, Chongqing, China; 2 Aier Academy of Ophthalmology, Central South University, Changsha, Hunan, China

**Keywords:** cavernous hemangiomas, minimally invasive surgery, multi-approach endoscopic strategy, navigation, orbital apex

## Abstract

**Objective:**

To evaluate the efficacy and safety of a tailored, navigation-assisted endoscopic surgical strategy for the removal of orbital apex cavernous hemangiomas, utilizing multiple approaches based on tumor location relative to the optic nerve.

**Methods:**

This retrospective case series analyzed ten consecutive patients who underwent surgical removal of orbital apex cavernous hemangiomas. Preoperative evaluation included MRI and/or CT scans to determine the tumor’s precise location. An individualized surgical plan was developed for each patient. Tumors medial to the optic nerve were removed via a navigation-assisted endoscopic transnasal approach (n=8). One tumor located superior to the optic nerve was resected via an endoscopic transsupraorbital (eyebrow) approach. One tumor lateral to the optic nerve was removed via an endoscopic lateral orbitotomy. We collected and analyzed data on patient demographics, tumor characteristics, surgical details, pre- and postoperative visual acuity (VA), intraocular pressure (IOP), eye motility, and complications.

**Results:**

All ten tumors were successfully resected, with pathology confirming cavernous hemangioma in all cases. The mean preoperative decimal VA was 0.304 ± 0.218, which significantly improved to 0.546 ± 0.314 at the 3-month postoperative follow-up (p = 0.0174, paired t-test). The median preoperative decimal VA was 0.25 (IQR, 0.1-0.48), which significantly improved to 0.55 (IQR, 0.3-0.9) at the 3-month postoperative follow-up (p = 0.0117, Wilcoxon signed-rank test). Sixty percent of patients (n=6) experienced a significant improvement in VA, while 40% (n=4) remained stable. No patients experienced a worsening of vision. Transient postoperative diplopia or ophthalmoplegia occurred in 80% of patients (n=8), but all resolved completely by the final follow-up, with all patients achieving normal eye motility. There were no cases of permanent vision loss, cerebrospinal fluid leakage, or other major complications.

**Conclusion:**

Based on strict, anatomy-driven patient selection, a tailored surgical strategy for orbital apex cavernous hemangiomas, utilizing navigation-assisted endoscopic approaches, is an effective and safe treatment modality for well-circumscribed lesions. This individualized approach maximizes tumor accessibility, minimizes surgical trauma, and leads to excellent visual outcomes with a low risk of permanent complications.

## 1 Introduction

Orbital apex cavernous hemangiomas are benign vascular malformations that, despite their slow growth, can cause significant morbidity due to the complex and crowded anatomy of the orbital apex (1). This conical space, bounded by the lesser and greater wings of the sphenoid and the orbital process of the palatine bone, houses a confluence of critical neurovascular structures, including the optic nerve, oculomotor nerve (III), trochlear nerve (IV), abducens nerve (VI), and the ophthalmic division of the trigeminal nerve (V1), as well as the ophthalmic artery and superior and inferior ophthalmic veins (2). Compression of these structures by an expanding lesion can lead to a constellation of debilitating symptoms, including progressive vision loss, visual field defects, proptosis, and ophthalmoplegia, collectively known as orbital apex syndrome (3–5).

The surgical management of these deep-seated lesions presents a formidable challenge. Historically, treatment relied on open transcranial approaches, such as the pterional or frontotemporal craniotomy, which often require extensive bone removal, significant brain retraction, and manipulation of the frontal or temporal lobes to gain access to the orbital apex (6). While these approaches can provide a wide field of view, they are associated with considerable morbidity, including the risk of cerebrospinal fluid (CSF) leakage, cerebral contusion, seizures, and, most critically, a high rate of iatrogenic injury to the optic nerve and other vital structures (7, 8). Several studies have reported that the risk of severe, permanent vision loss following transcranial orbitotomy can be as high as 18-24%, a devastating outcome for a benign condition (9, 10).

The last two decades have witnessed a paradigm shift in orbital surgery, driven by the advent of endoscopic techniques and intraoperative navigation systems (11). The endoscope, with its high-definition optics, angled lenses, and superior illumination, provides a magnified, panoramic view of the surgical field, allowing for meticulous dissection in confined spaces (12, 13). The endoscopic transnasal approach, in particular, has gained significant traction for lesions located medial to the optic nerve, offering a direct, minimally invasive corridor that avoids external incisions and minimizes manipulation of orbital tissues (12–14).

However, a single approach is not a panacea for all orbital apex tumors. The complex three-dimensional anatomy dictates that the optimal surgical corridor is entirely dependent on the tumor’s specific relationship to the optic nerve. A tumor located lateral to the nerve, for instance, is inaccessible via a purely transnasal route without risking injury to the nerve itself. This anatomical reality necessitates a more nuanced and flexible surgical philosophy.

The core innovation of this study lies in the systematic application and evaluation of a multi-approach, tailored surgical strategy. We hypothesize that by selecting the most direct endoscopic route based on the tumor’s precise location—transnasal for medial lesions, transsupraorbital (via eyebrow) for superior lesions, and lateral orbitotomy for lateral lesions—we can maximize surgical efficacy while simultaneously enhancing patient safety. This study presents a detailed analysis of the clinical outcomes of ten consecutive patients treated with this navigation-assisted, individualized endoscopic approach. Our aim is to provide robust, quantitative evidence for its efficacy, to delineate the specific advantages of each approach, and to establish a new benchmark for the safety profile achievable in the modern management of orbital apex cavernous hemangiomas.

## 2 Materials and methods

### 2.1 Patient selection

This retrospective study included ten consecutive patients diagnosed with orbital apex cavernous hemangiomas who underwent surgery at our institution between April 2022 and June 2025. The study was conducted in accordance with the principles of the Declaration of Helsinki and has been approved by Ethics Committee. Inclusion criteria were: (1) clinical symptoms and radiological findings (CT and/or MRI) consistent with a unilateral orbital apex tumor, suggestive of cavernous hemangioma; (2) tumor location causing or threatening visual impairment (e.g., documented decline in visual acuity, visual field defects, or significant proptosis); and (3) availability of complete pre- and postoperative clinical data, including detailed ophthalmological examinations and follow-up of at least 3 months. Patients with tumors extending significantly into the cranial cavity or those with contraindications to general anesthesia were excluded. All patients provided written informed consent after a comprehensive discussion of the surgical procedure, potential risks and benefits, and alternative treatment options such as observation or stereotactic radiosurgery.

### 2.2 Preoperative assessment and surgical planning

All patients underwent a comprehensive preoperative ophthalmological examination, which included best-corrected visual acuity (BCVA) testing using a Snellen chart, intraocular pressure (IOP) measurement via Goldmann applanation tonometry, Hertel exophthalmometry, and a detailed assessment of ocular motility and diplopia. Formal visual field testing was performed using Humphrey automated perimetry where patient cooperation allowed.

High-resolution magnetic resonance imaging (MRI) of the orbits with and without gadolinium contrast was the primary imaging modality. T1-weighted, T2-weighted, and fat-suppressed sequences were used to characterize the lesion. Cavernous hemangiomas typically appeared as well-circumscribed, encapsulated masses, isointense to muscle on T1-weighted images and markedly hyperintense on T2-weighted images, with progressive, heterogeneous enhancement following contrast administration. In some cases, high-resolution computed tomography (CT) with bone windows was also performed to evaluate the bony anatomy of the orbital apex, particularly the lamina papyracea and optic canal.

The cornerstone of our surgical planning was the meticulous analysis of the tumor’s three-dimensional anatomical relationship to the optic nerve on coronal and axial MRI scans. This relationship was the sole determinant for selecting the surgical approach.

### 2.3 Surgical techniques

All procedures were performed under general anesthesia by the same surgical team. An electromagnetic intraoperative navigation system (Medtronic StealthStation S8) was used in all cases. The system was registered using facial landmarks, providing real-time tracking of surgical instruments with sub-millimeter accuracy.

- Navigation-assisted endoscopic transnasal approach (n=8): This approach was selected for all tumors located primarily medial or inferomedial to the optic nerve. After induction of general anesthesia, the electromagnetic navigation system’s emitter and patient tracker were installed. The patient’s information was registered with a probe, and the accuracy of the navigation was verified before identifying the surgical instruments. The nasal mucosa was decongested with adrenaline-soaked cottonoids. The procedure was performed via a middle meatal approach. Under endoscopic guidance, the ethmoid and sphenoid sinuses were opened. The medial orbital wall (lamina papyracea) was thinned and removed to expose the periorbita and the orbital entrance of the optic nerve, providing direct access to the deep orbital apex. The tumor was precisely dissected under endoscopic view. The tumor, whether in the intraconal or extraconal space, was gradually separated from surrounding normal tissues, with maximal preservation of the optic nerve, ophthalmic artery, and other vital neurovascular structures, ultimately achieving complete tumor removal. The surgical cavity was inspected for hemostasis, and a small piece of absorbable gelatin sponge was placed over the orbital defect. This approach minimizes the risk of additional pressure on the optic nerve compared to a transconjunctival approach, making it particularly suitable for extraconal tumors. For intraconal tumors, retraction of the medial rectus muscle is required, which can be more challenging and may lead to postoperative muscle palsy.
- Navigation-assisted endoscopic transsupraorbital (eyebrow) approach (n=1): This approach was utilized for a tumor located directly superior to the optic nerve. Following navigation system setup, a 1.5–2 cm skin incision was made below the eyebrow using an electrosurgical knife. The skin, subcutaneous tissue, and orbital periosteum were incised in layers. The orbital septum was opened, and blunt dissection was performed towards the orbital apex. The navigation system was used to locate the tumor, which was found to be deep within the apex, with limited exposure. Under endoscopic guidance, blunt dissection continued, revealing a dark red, soft tumor, which was carefully separated from adherent surrounding tissues. The tumor was then grasped with an Allis clamp and removed completely. After ensuring hemostasis, the periosteum and muscle layers were sutured with 6–0 absorbable sutures, followed by continuous subcutaneous sutures. The eye was dressed with antibiotic ointment and a pressure bandage.
- Navigation-assisted endoscopic lateral orbitotomy (n=1): For the single tumor located lateral to the optic nerve, a modified Kronlein (lateral orbitotomy) approach was employed. A 1.5–2 cm incision was made in a skin crease of the lateral canthus. The lateral orbital rim was exposed, and a bone flap of the lateral orbital wall was temporarily removed using a piezoelectric saw. The periorbita was incised, and the space between the lateral and inferior rectus muscles was developed. With endoscopic visualization and navigation assistance, the tumor was identified and meticulously dissected away from the lateral aspect of the optic nerve and surrounding structures before being removed. The bone flap was repositioned and fixed with a titanium plate and three screws. The periosteum and muscle layers were sutured with 6–0 absorbable sutures, followed by continuous subcutaneous sutures. The eye was dressed with antibiotic ointment and a pressure bandage.

In all cases, the primary goal was complete, en bloc tumor resection while prioritizing the preservation of all neurovascular structures. Meticulous hemostasis was maintained throughout.

### 2.4 Postoperative care and follow-up

All patients were hospitalized for 5–7 days. Postoperative management included intravenous dexamethasone sodium phosphate to reduce inflammation, mannitol injection to alleviate edema, and a short course of antibiotics for infection prophylaxis. Patients who underwent lateral or transsupraorbital orbitotomy required pressure bandaging to prevent orbital hemorrhage. Patients were instructed to avoid nose-blowing and strenuous activity for two weeks. Follow-up examinations were conducted at 2 weeks, 1 month, 3 months, and then annually. The primary outcomes for this study were the changes in BCVA and ocular motility at the 3-month postoperative visit.

### 2.5 Statistical analysis

Visual acuity was converted from Snellen and counting fingers (CF) notation to a decimal equivalent for statistical analysis. Given the small sample size, a Shapiro-Wilk test was performed to assess the normality of the data distribution. As the data were not normally distributed (p < 0.05), pre- and postoperative continuous variables (VA, IOP) were compared using the non-parametric Wilcoxon signed-rank test. Descriptive statistics are presented as median and interquartile range (IQR), as well as mean and standard deviation (SD). A p-value of < 0.05 was considered statistically significant. All statistical analyses were performed using GraphPad Prism.

## 3 Results

### 3.1 Patient demographics and preoperative presentation

The study cohort consisted of 10 patients, comprising 8 females (80%) and 2 males (20%). The mean age at the time of surgery was 52.5 ± 12.8 years, with a range of 34 to 77 years. The left eye was affected in 6 cases (60%) and the right eye in 4 cases (40%). The most common presenting symptom was a gradual, progressive decline in visual acuity, reported by 7 of the 10 patients. Other presenting symptoms included proptosis (n=3), diplopia (n=2), and headache (n=1). In two cases, the tumor was an incidental finding on imaging performed for other reasons.

The mean preoperative decimal visual acuity was 0.304 ± 0.218, with a median of 0.25 (IQR, 0.1-0.48). Preoperative vision ranged from 0.6 to as low as counting fingers at 10 cm (decimal equivalent 0.01). The mean preoperative intraocular pressure was 14.3 ± 3.3 mmHg. The mean preoperative exophthalmos was 2.0 ± 1.3 mm (range, 1–5 mm). Two patients (P7 and P10) presented with preoperative ophthalmoplegia and diplopia. Detailed demographic and preoperative clinical data for each patient are presented in **Table 1**.

**Table 1:** Patient demographics and preoperative clinical data.

Patient ID	Age (years)	Gender	Affected eye	Pre-op VA	Pre-op IOP (mmHg)	Pre-op exophthalmos (mm)	Tumor location	Tumor size
P1	45	Female	Right	0.12	13	3	Medial to ON	1.6*1.2cm
P2	77	Female	Left	0.3	13	2	Medial to ON	1.5*1.6cm
P3	38	Female	Left	0.3	18	3	Medial to ON	1.5*1.6cm
P4	66	Female	Left	0.6	13	2	Medial to ON	1.0*0.8cm
P5	57	Female	Right	0.06	10	1	Medial to ON	2.0*1.5cm
P6	54	Female	Left	CF at 10cm	16	1	Medial to ON	1.0*1.5cm
P7	53	Female	Left	0.4	17	1	Medial & Superior to ON	1.2*1.2cm
P8	45	Male	Right	0.5	20	1	Lateral to ON	1.0*0.5cm
P9	34	Female	Left	0.6	10	1	Medial to ON	1.0*0.5cm
P10	56	Male	Right	0.15	13	5	Medial to ON	2*2.5cm

### 3.2 Tumor characteristics and surgical approach

All tumors were located in the orbital apex. Based on their relationship to the optic nerve, 8 tumors (80%) were classified as medial, 1 (10%) as superior, and 1 (10%) as lateral. The mean maximum tumor diameter was 1.50 ± 0.49 cm (range, 1.0 to 2.5 cm).

The surgical approach was strictly tailored to the tumor’s location. The 8 medial tumors were all resected via the endoscopic transnasal approach. The superior tumor was resected via the endoscopic transsupraorbital approach, and the lateral tumor was resected via the endoscopic lateral orbitotomy. The mean operative time for the entire cohort was 92.2 ± 39.5 minutes (range, 42–162 minutes). All 10 tumors were successfully resected in their entirety, with no gross residual tumor observed at the conclusion of surgery. Histopathological examination confirmed the diagnosis of cavernous hemangioma in all 10 cases.

### 3.3 Postoperative visual and functional outcomes

A statistically significant improvement in visual acuity was observed for the cohort. The median decimal visual acuity improved from 0.25 (IQR, 0.1-0.48) preoperatively to 0.55 (IQR, 0.3-0.9) at the 3-month postoperative visit (p = 0.0117, Wilcoxon signed-rank test). The mean decimal VA improved from 0.304 ± 0.218 to 0.546 ± 0.314.

Analyzing the outcomes on an individual basis, six patients (60%) experienced a significant improvement in vision (defined as an improvement of two or more Snellen lines). For example, Patient P1 improved from 0.12 to 0.8, and Patient P8 improved from 0.5 to 1.0. Four patients (40%) had their vision remain stable, with no significant change from their preoperative baseline. This included Patient P6, who presented with very poor vision (CF at 10cm) and optic atrophy; her vision improved slightly to CF at 40cm, but the primary goal of preventing complete blindness was achieved. Crucially, no patient in this series experienced a permanent decline in visual acuity as a result of the surgery.

There was no statistically significant change in intraocular pressure from the preoperative mean of 14.3 ± 3.3 mmHg to the postoperative mean of 13.7 ± 3.3 mmHg (p = 0.5313, Wilcoxon signed-rank test). Proptosis, where present, resolved in all patients. Surgical details and postoperative outcomes are summarized in **Table 2**.

**Table 2:** Surgical details and postoperative outcomes.

Patient ID	Surgical approach	Operative time (min)	Post-op VA (3 months)	VA change	Post-op IOP (mmHg)	Eye movement at final F/U	Diplopia at final F/U	Pathology
P1	Endoscopic Transnasal	92	0.8	Improved	11	Normal	nan	Cavernous Hemangioma
P2	Endoscopic Transnasal	70	0.4	Improved	17	Normal	nan	Cavernous Hemangioma
P3	Endoscopic Transnasal	154	0.4	Improved	18	Normal	nan	Cavernous Hemangioma
P4	Endoscopic Transnasal	105	0.8	Improved	16	Normal	nan	Cavernous Hemangioma
P5	Endoscopic Transnasal	55	0.12	Stable	13	Normal	nan	Cavernous Hemangioma
P6	Endoscopic Transnasal	162	CF at 40cm	Stable	11	Normal	nan	Cavernous Hemangioma
P7	Endoscopic Transsupraorbital	65	0.5	Stable	15	Normal	nan	Cavernous Hemangioma
P8	Endoscopic Lateral Orbitotomy	92	1.0	Improved	17	Normal	nan	Cavernous Hemangioma
P9	Endoscopic Transnasal	85	0.6	Stable	9	Normal	nan	Cavernous Hemangioma
P10	Endoscopic Transnasal	42	0.8	Improved	10	Normal	nan	Cavernous Hemangioma

### 3.4 Complications and safety profile

The surgical procedures were well-tolerated, with no major intraoperative or immediate postoperative events. There were no instances of intraoperative CSF leakage, major hemorrhage requiring blood transfusion, or iatrogenic injury to the globe or optic nerve.

The most common postoperative issue was transient diplopia and/or ophthalmoplegia, which occurred in 8 of the 10 patients (80%). This was most frequently observed in patients who underwent the transnasal approach and was typically characterized by a temporary adduction deficit of the affected eye, consistent with neuropraxia of the medial rectus muscle or its nerve supply from retraction during surgery. For instance, Patient P1 developed a postoperative adduction deficit that resolved within two months. Patient P7, who had a complex history including a prior failed surgery elsewhere and presented with preoperative ophthalmoplegia, underwent a transsupraorbital approach and experienced postoperative ptosis and an elevation deficit, both of which resolved completely by 3 months.

The key finding regarding complications is that all instances of postoperative ophthalmoplegia and diplopia were transient. By the final follow-up visit (ranging from 3 to 12 months), all 10 patients (100%) had regained full and normal ocular motility with complete resolution of any diplopia. The safety profile was excellent, with a 0% rate of permanent neurological deficits, including permanent vision loss or permanent cranial nerve palsy.

### 3.5 Illustrative case vignettes

Case 1: Patient P2 (Endoscopic Transnasal Approach)

**Figure 1:**
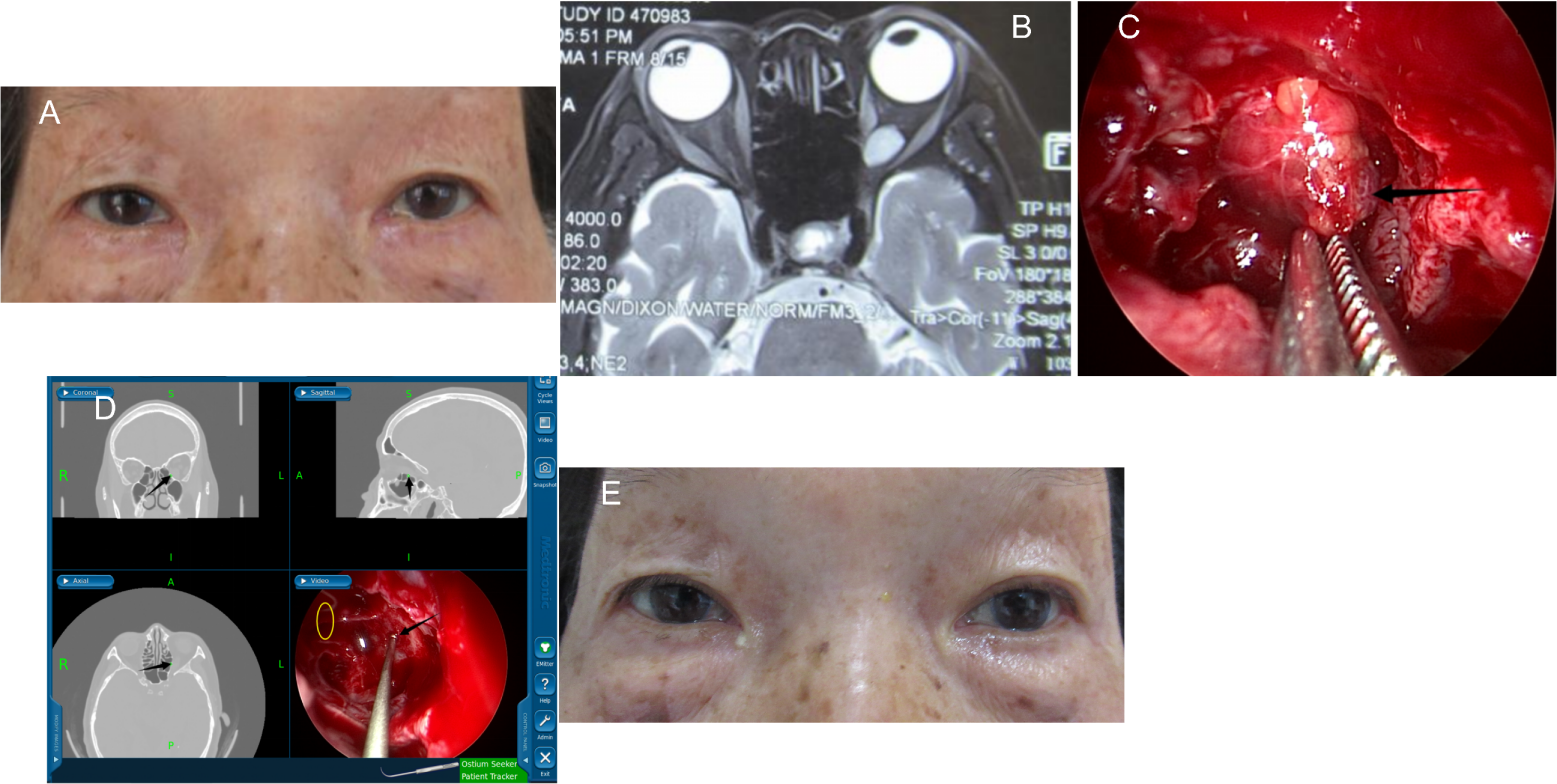
Patient P2 - endoscopic transnasal resection of left orbital apex cavernous hemangioma. **(A)** Preoperative facial appearance. **(B)** Axial MRI showing the left orbital apex cavernous hemangioma. **(C)** Endoscopic transnasal surgical removal of the tumor (black arrow). **(D)** Intraoperative navigation localization of the tumor (black arrow), skull base (yellow circle). **(E)** Postoperative facial appearance.

A 77-year-old female presented with a gradual decline in vision in her left eye to 0.3. MRI revealed a 1.5 x 1.6 cm well-circumscribed mass in the left orbital apex, medial to the optic nerve. A navigation-assisted endoscopic transnasal approach was performed. The tumor was completely resected, decompressing the optic nerve. Postoperatively, her vision improved to 0.4 at the 3-month follow-up, and she experienced no significant complications (**Figure 1**).

Case 2: Patient P7 (Endoscopic Transsupraorbital Approach)

**Figure 2:**
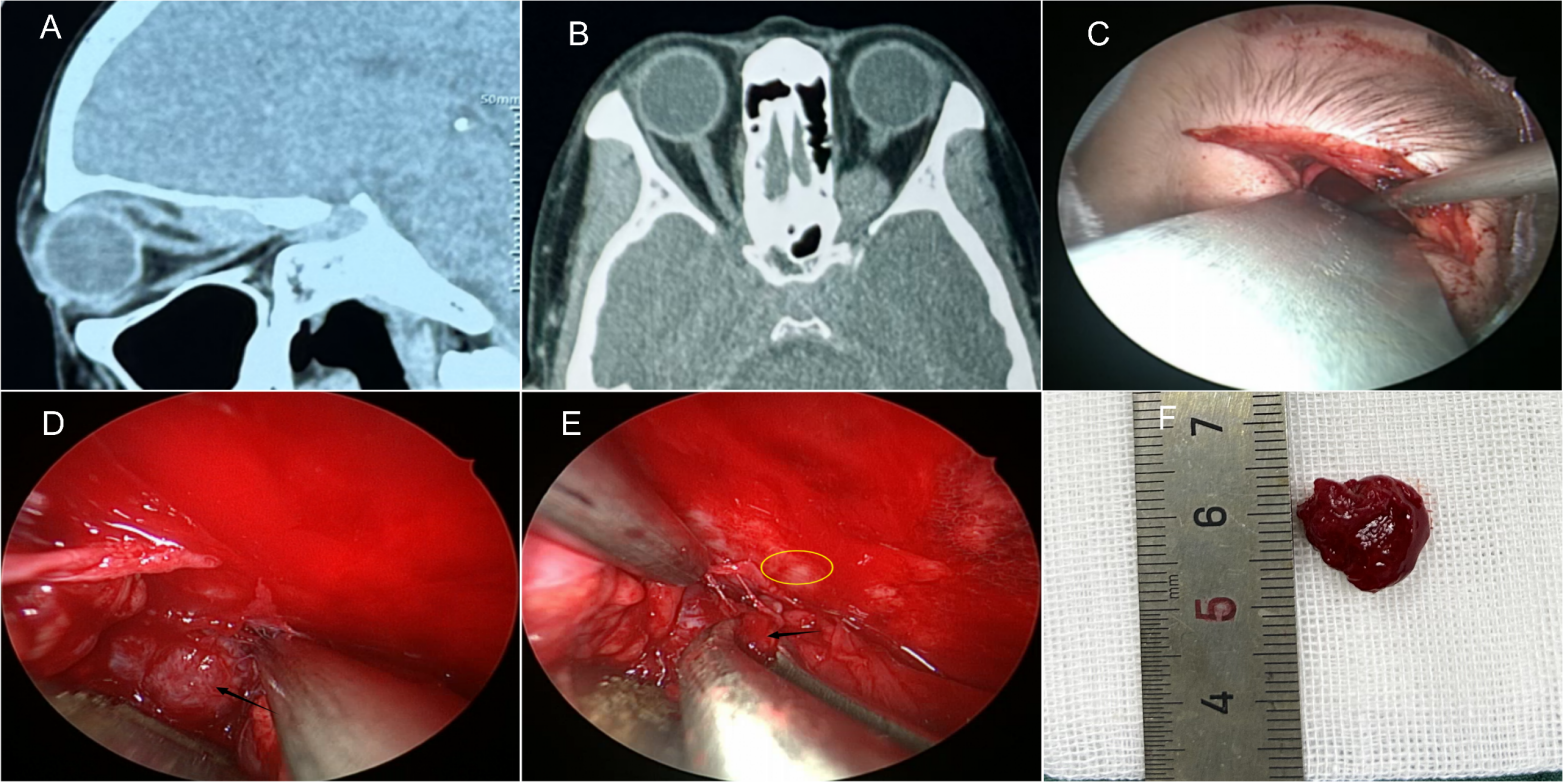
Patient P7 - Endoscopic Transsupraorbital Resection of Left Orbital Apex Cavernous Hemangioma. **(A)** Sagittal MRI showing the left orbital apex cavernous hemangioma, with the tumor located superior to the optic nerve. **(B)** Axial MRI showing the left orbital apex cavernous hemangioma. **(C)** Endoscopic transsupraorbital surgical approach with skin incision below the eyebrow. **(D)** Endoscopic-assisted exposure of the deep orbital tumor (black arrow). **(E)** Endoscopic-assisted grasping and complete removal of the tumor (black arrow), deep lateral orbital wall(yellow circle). **(F)** Excised tumor specimen.

A 53-year-old female presented with a complex history, including a prior failed open orbital exploration at another institution for a deep orbital tumor. She presented to us with a visual acuity of 0.4 in the left eye, significant preoperative diplopia, and restricted eye movement in all directions. MRI confirmed a 1.2 x 1.2 cm mass in the orbital apex, located superior to the optic nerve. Due to the superior location and the presence of scar tissue from the previous surgery, an endoscopic transsupraorbital (eyebrow) approach was selected. The surgery, lasting 65 minutes, involved careful dissection through the scarred tissue to identify and resect the tumor from the superior aspect of the optic nerve. Postoperatively, she had transient ptosis and a persistent, though improved, upgaze restriction. By her 1-month follow-up, her vision had improved to 0.5, and by 3 months, the ptosis had completely resolved, and her eye movements had returned to normal. This case highlights the utility of this approach for superiorly located lesions, even in a re-operative setting (**Figure 2**).

Case 3: Patient P8 (Endoscopic Lateral Orbitotomy)

**Figure 3:**
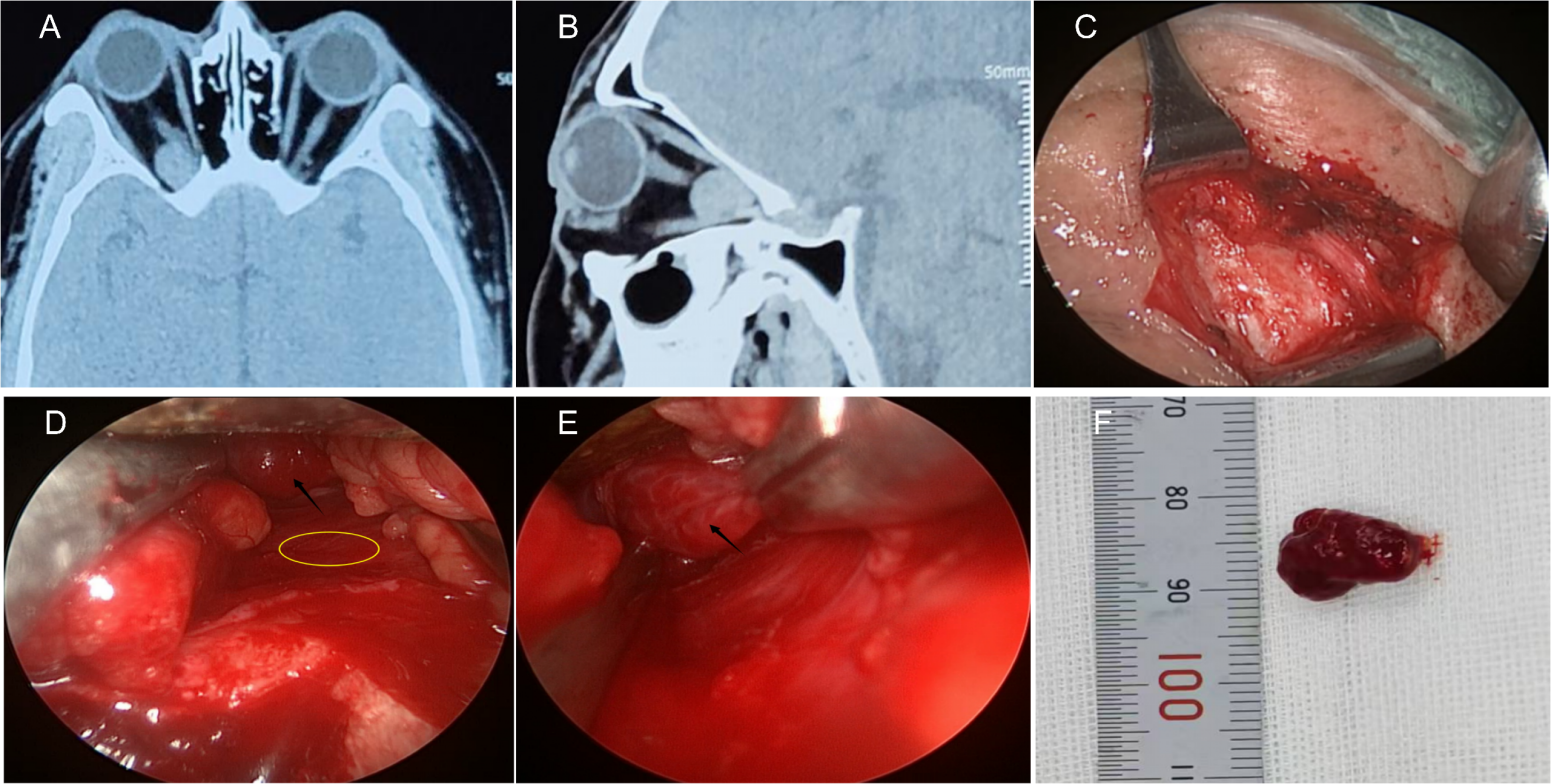
Patient P8 - Endoscopic Lateral Orbitotomy Resection of Right Orbital Apex Cavernous Hemangioma. **(A)** Axial MRI showing the right orbital apex cavernous hemangioma, with the tumor located lateral to the optic nerve. **(B)** Sagittal MRI showing the right orbital apex cavernous hemangioma. **(C)** Endoscopic lateral orbitotomy surgical approach with lateral skin incision exposing the lateral orbital wall. **(D, E)** Endoscopic-assisted exposure of the deep orbital tumor (black arrow), skull base (yellow circle). **(F)** Excised tumor specimen.

A 45-year-old male presented with a decline in vision in his right eye to 0.5. MRI revealed a 1.0 x 0.5 cm mass in the right orbital apex, lateral to the optic nerve. An endoscopic-assisted lateral orbitotomy was performed. The tumor was completely resected. Postoperatively, his vision improved dramatically to 1.0, and he experienced no complications (**Figure 3**).

## 4 Discussion

The surgical treatment of orbital apex cavernous hemangiomas has historically been fraught with peril, balancing the need for tumor removal against the high risk of devastating functional loss. The findings of this study provide compelling evidence that a modern, technologically augmented, and anatomically logical surgical strategy can dramatically shift this risk-benefit calculus in favor of the patient. Our 100% tumor resection rate, statistically significant improvement in visual function, and, most importantly, 0% rate of permanent complications underscore the value of a tailored, multi-approach endoscopic and navigation-assisted methodology.

The established dogma in neuro-ophthalmic surgery is that the approach must be tailored to the pathology. Our study takes this principle a step further by demonstrating a systematic, anatomy-based algorithm for selecting among multiple minimally invasive corridors. The traditional debate often pitted a single transcranial approach against a single transorbital one. We propose a more granular paradigm where the choice is between multiple endoscopic-assisted routes, each with specific advantages. The transnasal route is not merely an alternative; it is the optimal approach for medial lesions, just as the lateral orbitotomy is optimal for lateral ones. This is not just a matter of surgeon preference but a conclusion dictated by biomechanics and the goal of minimizing retraction on the optic nerve. Our results validate this principle. For the 80% of tumors located medial to the optic nerve, the endoscopic transnasal approach proved to be an elegant and effective solution, leveraging the natural corridor of the paranasal sinuses to arrive directly at the lesion without an external incision or orbital rim osteotomy (12–14). For the superiorly located tumor, the transsupraorbital approach provided direct access, while the lateral orbitotomy was the logical choice for the laterally-positioned tumor. This study is one of the first to systematically report on the combined use of these three distinct, anatomy-driven endoscopic approaches within a single coherent treatment strategy for orbital apex hemangiomas.

The success of our approach is not due to the choice of corridor alone, but to the powerful synergy between endoscopic visualization and computer-assisted navigation. The endoscope transforms the deep, dark confines of the orbital apex into a well-lit, magnified surgical field, allowing the surgeon to “look around corners” and ensure complete dissection of the tumor capsule from the delicate optic nerve sheath (15). Intraoperative navigation acts as the surgeon’s “GPS”, providing a crucial layer of safety and confidence by allowing for the precise localization of the tumor and the real-time identification of critical structures. This accuracy allows for a more focused and efficient procedure, reflected in our mean operative time of 92.2 minutes, which is considerably shorter than many reported times for open transcranial approaches.It is important to note that the successful implementation of this multi-approach strategy is dependent on the surgical team’s expertise in advanced endoscopic orbital surgery, which involves a significant learning curve.

However, it is crucial to contextualize the indications for this endoscopic strategy. Its success is predicated on the benign, well-encapsulated nature of cavernous hemangiomas. For more complex pathologies, such as those that are invasive, multicompartmental, or recurrent, purely endoscopic approaches may be insufficient. In such scenarios, traditional open microsurgical craniotomies, including the pterional, frontotemporal, or modified orbitozygomatic approaches, remain indispensable (16, 17). These conventional techniques provide a wider surgical field, superior proximal and distal control of major vascular structures, and greater flexibility for extensive reconstruction, which are critical for managing tumors that infiltrate surrounding tissues or encase neurovascular structures (18). Therefore, the modern orbital surgeon’s armamentarium must include both advanced endoscopic techniques for well-selected cases and the proven efficacy of open approaches for more complex challenges.

When compared to traditional “gold standard” open approaches, such as transcranial craniotomies, our minimally invasive strategy offers several potential advantages. By avoiding large skin incisions, extensive bone removal, and brain retraction, our approaches significantly reduce surgical trauma, leading to shorter hospital stays and faster recovery. Most critically, by creating a direct, tailored corridor to the tumor, we minimize manipulation of the optic nerve and surrounding critical structures. This is reflected in our 0% rate of permanent vision loss, a stark contrast to the rates of 1.8% to as high as 24% reported for some traditional transcranial series (9, 10). While our approach is specifically tailored to cavernous hemangiomas and may not be suitable for all orbital apex pathologies, it represents a significant advancement in safety and efficacy for this specific indication.

The clinical outcomes presented in this study set a new benchmark for safety and efficacy. The 0% rate of permanent vision loss is a stark contrast to the rates of 1.8% or higher reported for some traditional transcranial series (9, 10). This profound difference highlights the fundamental advantage of avoiding brain retraction and extensive orbital disruption. Our study’s 80% rate of transient ophthalmoplegia warrants further discussion. While this figure may seem high, it must be interpreted in the context of the pathology’s location. The orbital apex is arguably the most densely packed functional real estate in the human body. Any surgical manipulation, no matter how gentle, is likely to cause temporary neuropraxia of the fine motor nerves to the extraocular muscles. The critical differentiating factor between our approach and more aggressive open approaches is the nature of the injury. Our results, with 100% recovery of function, strongly suggest that the injury is a transient, stretch-related neuropraxia from which the nerve can fully recover. In contrast, the direct trauma, devascularization, or contusion associated with larger craniotomies can lead to permanent axonotmetic injury and irreversible functional loss. Therefore, we posit that transient, fully recoverable ophthalmoplegia is an acceptable and expected sequela of successful orbital apex decompression, and its high incidence should not be misinterpreted as a high complication rate in the traditional sense.

While complete surgical excision remains the gold standard for symptomatic orbital apex cavernous hemangiomas, several alternative and less invasive treatment modalities have been explored in recent years. For certain vascular malformations, intralesional sclerotherapy with agents like bleomycin or its analogue, pingyangmycin, has shown promise (19, 20). These agents induce inflammation and fibrosis within the lesion, leading to shrinkage. Studies by Yue et al. and Jia et al. have demonstrated the efficacy of pingyangmycin for low-flow orbital venous malformations, including cavernous hemangiomas, reporting significant volume reduction (19, 20). However, the use of sclerotherapy in the orbital apex is fraught with risk. The inflammatory response, while therapeutic, is largely uncontrolled and can extend to adjacent critical structures, potentially causing irreversible damage to the optic nerve or cranial nerves (21). Furthermore, sclerotherapy does not provide immediate mass effect reduction, and the fibrotic remnant can still cause compression. Given these risks, sclerotherapy is generally reserved for more diffuse, low-flow venous malformations rather than the well-encapsulated cavernous hemangiomas, especially in the perilous environment of the orbital apex.

Another non-invasive alternative is stereotactic radiosurgery, such as Gamma Knife radiosurgery (GKRS). Several studies have reported on the use of GKRS for orbital cavernous hemangiomas, demonstrating high rates of tumor control and, in many cases, improvement in clinical symptoms (22–24). A recent meta-analysis by Punukollu et al. found that GKRS resulted in visual acuity improvement in 80% of cases and tumor reduction in 77%, with a low complication rate (24). However, GKRS has several drawbacks compared to surgery. Firstly, its effect is delayed, and it does not provide the immediate optic nerve decompression that is often required in patients with acute or progressive vision loss. Secondly, there is a small but significant risk of radiation-induced optic neuropathy, a delayed and often irreversible complication (25). Thirdly, radiosurgery does not provide a histopathological diagnosis, which, while the diagnosis of cavernous hemangioma is often clear on MRI, can never be 100% certain without tissue confirmation. Therefore, while GKRS may be a viable option for patients who are poor surgical candidates, or perhaps for small, asymptomatic tumors, we maintain that for healthy patients with symptomatic, surgically accessible lesions, complete surgical excision provides the most definitive and immediate treatment.

This study has several important limitations. First, the retrospective, single-center design and the small cohort size (n=10) limit the generalizability of our findings. Although orbital apex cavernous hemangiomas are rare, a larger, multi-center study would be needed to confirm our results. Second, the absence of a direct control group undergoing traditional open surgery prevents a definitive conclusion regarding the superiority of our approach; our comparisons are based on historical literature, which may be subject to selection bias. Third, the median follow-up period is relatively short for these slow-growing tumors, which makes it difficult to assess long-term recurrence rates or the incidence of late complications. Finally, while gross total resection was achieved based on intraoperative assessment, the lack of routine postoperative contrast-enhanced MRI to objectively confirm the absence of residual tumor is a limitation. Future prospective studies should incorporate standardized postoperative imaging to definitively verify the extent of resection.A larger, multi-center, prospective study would be the ideal next step to validate our findings and to establish this tailored, multi-approach strategy as a widespread standard of care. Looking forward, the principles outlined in this study can be extended and refined. The integration of robotic surgery platforms with endoscopic instruments could further enhance dexterity and tremor filtration, potentially reducing even the rate of transient neuropraxia. Augmented reality overlays, projecting the segmented tumor and optic nerve from the preoperative MRI directly into the endoscopic view, could provide an even more intuitive and immersive navigational experience. Our study provides a robust baseline of safety and efficacy upon which these future technological innovations can be built.

## 5 Conclusion

Based on strict, anatomy-driven patient selection, a tailored surgical strategy for orbital apex cavernous hemangiomas, utilizing navigation-assisted endoscopic approaches, is an effective and safe treatment modality for well-circumscribed lesions. This individualized approach maximizes tumor accessibility, minimizes surgical trauma, and leads to excellent visual outcomes with a low risk of permanent complications. The choice of a specific endoscopic corridor—be it transnasal, transsupraorbital, or lateral—should be dictated by the tumor’s precise anatomical relationship to the optic nerve. While this strategy represents a significant advancement for select cases, complex or invasive lesions may still necessitate traditional open microsurgical approaches.

## Data Availability

The original contributions presented in the study are included in the article/supplementary material. Further inquiries can be directed to the corresponding authors.
